# Effects of ECM Components on Periodontal Ligament Stem Cell Differentiation Under Conditions of Disruption of Wnt and TGF-β Signaling Pathways

**DOI:** 10.3390/jfb16030094

**Published:** 2025-03-09

**Authors:** Alla V. Kuznetsova, Olga P. Popova, Tamara I. Danilova, Andrey V. Latyshev, Oleg O. Yanushevich, Alexey A. Ivanov

**Affiliations:** 1 Laboratory of Molecular and Cellular Pathology, Russian University of Medicine (Formerly A.I. Evdokimov Moscow State University of Medicine and Dentistry), Ministry of Health of the Russian Federation, Bld 4, Dolgorukovskaya Str, 127006 Moscow, Russia; avkuzn@list.ru (A.V.K.); petrovnapopova@rambler.ru (O.P.P.); danilova.tam@yandex.ru (T.I.D.); doctor.latyshev@gmail.com (A.V.L.); 2 Koltzov Institute of Developmental Biology, Russian Academy of Sciences, 26 Vavilov Str, 119334 Moscow, Russia; 3 Department of Periodontology, Russian University of Medicine (Formerly A.I. Evdokimov Moscow State University of Medicine and Dentistry), Ministry of Health of the Russian Federation, Bld 4, Dolgorukovskaya Str, 127006 Moscow, Russia; olegyanushevich@me.com

**Keywords:** decellularized extracellular matrix, collagen I hydrogel, hyaluronic acid, fibronectin, laminin, periodontal ligament stem cell differentiation, TGF-β signaling, Wnt/β-catenin signaling

## Abstract

Periodontitis is accompanied by inflammation that causes dysregulation of the Wnt/β-catenin and TGF-β signaling pathways. This leads to a violation of the homeostasis of periodontal tissues. Components of the extracellular matrix (ECM) are an important part of biomaterials used for the repair of periodontal tissue. The purpose of this study was to evaluate the components of the effect of ECM (hyaluronic acid (HA), fibronectin (Fn), and laminin (Lam)) on the osteogenic and odontogenic differentiation of periodontal ligament stem cells (PDLSCs) in the collagen I hydrogel under conditions of disruption of the Wnt/β-catenin and TGF-β signaling pathways. The study showed that the addition of components of the ECM restored the expression of odontogenic markers in PDLSCs, which was absent during inhibition of the canonical Wnt signaling pathway, and their multidirectional effect on the secretion of transforming growth factor-β1 (TGF-β1) and bone morphogenetic protein 2 (BMP-2). Fn and Lam suppressed the expression of odontogenic markers in PDLSCs against the background of inhibition of the TGF-β signaling pathway. The addition of HA under the conditions of the TGF-β signaling pathway improved BMP-2 secretion, preserving odontogenic differentiation. Thus, our results demonstrated that disruption of the Wnt/β-catenin and TGF-β signaling pathways causes disorders in the differentiation of PDLSCs, preventing the regeneration of periodontal tissues. This should be taken into account when developing multicomponent scaffolds that recapitulate the ECM microenvironment at endogenic regeneration of the periodontium. Inclusion of hyaluronic acid as one of these components may enhance the therapeutic effect of such biomaterials.

## 1. Introduction

Inflammation that induces damage to periodontal tissues leads to a disruption of homeostasis that supports their normal functioning [1,2]. Restoration of mechanisms aimed at maintaining the homeostasis of periodontal tissues is a key approach that ensures their natural regeneration from the point of view of regenerative dentistry. The Wnt/β-catenin and TGF-β signaling pathways are involved in both the development and regulation of periodontal tissue function [3,4,5,6].

The Wnt signaling pathway plays an important role in periodontal tissue homeostasis, periodontal cell function, and periodontal progression [7,8]. The Wnt signaling pathway regulates the proliferation and differentiation of periodontal ligament stem cells (PDLSCs) and is involved in alveolar process, the formation and orientation of the periodontal ligament (PDL), and cementum regeneration. Furthermore, data from in vitro studies suggest that each strand of the Wnt pathway may have different functions: the canonical Wnt/β-catenin pathway correlates with osteogenic differentiation, and the noncanonical Wnt pathway may inhibit this differentiation [9]. Modulators targeting Wnt signaling are considered adjuvant therapy in the treatment of periodontitis [10].

The TGF-β superfamily, including TGF-β1 and BMP, plays a key role in many processes of periodontal tissue development. Matrix-associated forms of TGF-β are present in the alveolar bone and connective tissue of the periodontium and are reported to be the most significant cytokine distributed throughout the PDL. Interestingly, the level of TGF-β expression in PDL is higher than in pulp or alveolar bone [11]. This indicates the active participation of TGF-β in maintaining homeostasis and regeneration of periodontal tissues. However, the function of endogenous TGF-β in PDL cells is not fully understood. Although the signaling activity of the TGF-β pathway determines the strength and duration of the response, the nature of this response depends on many microenvironmental factors (type and state of target cells and ECM type), as well as the presence of other signaling pathways that modify the response [11].

The transmission of Wnt and TGF-β signaling has a dual effect on cementogenic differentiation due to the difficulty of crosslinking to multiple signaling pathways. When PDLSCs are at different stages of differentiation or in different microenvironments, the role of Wnt and TGF-β signaling can be controversial. Furthermore, there is little or no information available on the effects of the Wnt and TGF-β signaling pathway on the PDL and cementum in an inflammatory environment that induces its dysregulation [10,12].

Biomaterials are widely used in the repair of the dental microenvironment by providing conditions for the migration and expansion of resident stem cells and to support their self-renewal and appropriate cell differentiation [13]. They should mimic the structure and ECM composition of periodontal tissues, recreating their microenvironment. The components of ECM are an important part of functional biomaterials that are used to induce phenotypic plasticity and differentiate resident periodontal tissue stem cells to restore them [14]. They are a reservoir of matrix-associated pro-inflammatory cytokines, growth factors of the TGF-β family, including several BMPs, and angiogenic growth factors, such as vascular endothelial growth factor, which are required to achieve osteoinduction by regulating different phases of regeneration [15]. Previously, we showed that the combination of the decellularized tooth matrix (dTM) and the decellularized periodontal ligament (dPDL) in a collagen I hydrogel induces spontaneous differentiation of PDLSCs in the osteogenic and odontogenic directions, and the addition of fibronectin (Fn) induces differentiation of PDLSCs into odontoblast- and osteoblast-like cells [16]. However, the effect of this bioengineered construct and the exogenous components of the ECM on the differentiation of PDLSCs under conditions of dysregulation of signaling pathways is unknown.

The purpose of this study was to evaluate the effect of ECM components on the osteogenic and odontogenic differentiation of PDLSCs in the collagen I hydrogel with decellularized matrices under the conditions of disruption of Wnt/β-catenin and TGF-β signaling pathways.

## 2. Materials and Methods

### 2.1. Preparation of Decellularized Matrices

Healthy teeth (1–3 molars) were used to make bioengineered constructs. The teeth were obtained with the written consent of the patients. The planned tooth extraction was performed during orthodontic treatment. The procedures were carried out according to the guidelines established by the Ethical Committee of the Russian University of Medicine (code 03/20 from 18 March 2020). Under aseptic conditions, PDL tissues were separated from the surface of the middle third of the tooth root, cut into strips (0.5 to 0.7 mm), and stored at -20 °C. The teeth were dissected at the cement–enamel junction site. The remaining tooth roots were used to form particles 1–2 mm thick with an electric mill (Bosch MKM 6000, Görlingen, Germany). Next, to obtain decellularized matrices, PDL strips and tooth particles were subjected to sequential treatment with chemical detergents (1% SDS solution and Triton X-100 solution) and enzymes (20 μg/mL DNase solution) (all reagents from Sigma-Aldrich, St. Louis, MO, USA) using procedures previously described (farther–dPDL and dTM, respectively) [17].

### 2.2. ECM Components

The components of ECM at the final concentration (sodium hyaluronate (HA; 1 mg/mL, Dongkook Pharmaceutical Co., Ltd., Seoul, Korea), fibronectin (Fn; 10 μg/mL, Gibco™ 33016015), and laminin (Lam; 1 μg/mL, Gibco™ 23017015)) were added separately to the collagen I hydrogel.

### 2.3. Signaling Pathway Inhibition

To inhibit the Wnt/β-catenin signaling pathway, a small molecule IWP-2 (STEMCELL Technologies, New York, NY, USA) was used at a concentration of 1.1 μg/mL, which blocks cell proliferation and differentiation along the canonical pathway. To block the TGF-β signaling pathway, a small molecule A 83-01 was used at a concentration of 2.1 μg/mL, which inhibits type I receptor TGF-β, but does not affect the receptors of BMPs. Stock solutions of inhibitors have been dissolved in 0.05% DMSO (Sigma-Aldrich, St. Louis, MO, USA) according to the manufacturer’s protocol.

### 2.4. Preparation of Bioengineered Constructs in Collagen I Hydrogel

Collagen I hydrogel (Imtek, Russia) and PDLSCs were received according to the procedures previously described [17]. PDLSCs were obtained from PDL tissues by enzyme extraction and cultured in DMEM-GlutaMAX growth medium supplemented with 2 mM essential amino acids, 15% fetal bovine serum (FBS), and antibiotics (100 U/ml penicillin and 100 µg/mL streptomycin) (all reagents from Gibco, USA) at 37 °C, 5% CO_2_.

The neutralized collagen I hydrogel with an embedded combination of dTM and dPDL, the suspension of PDLSCs (at a final concentration of 0.5 × 10^6^ cells/mL), and the ECM component were placed in the wells of a 12-well plate in a volume of 1.5 mL per well and left to polymerize at 37 °C, 5% CO_2_ for 60 min. After collagen polymerization, prewarmed complete DMEM-GlutaMAX (Gibco) with 10% FBS containing the signal pathway inhibitors were added to bioengineered constructs. Cells were cultured for 14 days with a change of medium every 3 days with the addition of DMSO or inhibitors.

As a control, PDLSCs were cultured with a combination of decellularized matrices in a collagen I hydrogel in a growth medium with the addition of 0.05% DMSO, which was used to prepare inhibitor stock solutions without (group 1) or with the addition of ECM components (groups 2–4). In the experiment, group 1 (without inhibitors with 0.05% DMSO) was compared with groups 5 (inhWn) and 6 (inhTGFβ), group 2 with groups 7 and 10, group 3 with groups 8 and 11, and group 4 with groups 9 and 12 respectively, as follows:

(1) Col I + dECM + DMSO (DMSO);
(2) Col I + dECM + DMSO + HA (DMSO+HA);
(3) Col I + dECM + DMSO + Fn (DMSO+Fn);
(4) Col I + dECM + DMSO + Lam (DMSO+Lam);
(5) Col I + dECM + inhWnt (inhWnt);
(6) Col I + dECM + inhTGF-β (inhTGFβ);
(7) Col I + dECM + inhWnt + HA (inhWnt+HA);
(8) Col I + dECM + inhWnt + Fn (inhWnt+Fn);
(9) Col I + dECM + inhWnt + Lam (inhWnt+Lam);
(10) Col I + dECM + inhTGFβ + HA (inhTGFβ+HA);
(11) Col I + dECM + inhTGFβ + Fn (inhTGFβ+Fn);
(12) Col I + dECM + inhTGFβ + Lam (inhTGFβ+Lam).

### 2.5. Histological and Immunohistochemical Examination

The 3D cultured cells were fixed with 10% neutral formalin on day 14. Next, the samples were decalcified, embedded in paraffin, and the histological specimens were prepared according to standard procedures.

For the immunohistochemistry, deparaffinized sections were incubated with antibodies to osteopontin (OPN, ab218237, Abcam, Newark, DE, USA), osteocalcin (OC, ab198228, Abcam), dentin sialophosphoprotein (DSPP, ab216892, Abcam), dentin matrix acidic phosphoprotein 1 (DMP-1, HPA037465, Sigma-Aldrich, St. Louis, MO, USA), and alkaline phosphatase (ALP, ab216892, Abcam). Before immunohistochemical staining, antigen recovery was performed using Dako PT Link (Dako, Glostrup, Denmark A/S) at 97 ° C for 20 min. The unmasking was performed under low pH using EnVison FLEX Target Retrieval Solution (Dako, Denmark A/S). Endogenous peroxidase and host IgG were blocked, and primary antibodies at the appropriate dilution were incubated for 12 h at 4 °C. The EnVision FLEX detection system (Dako, Denmark A/S) with the chromogen 2, 3-diaminobenzidine DAB (DAB Chromogen Solution, Dako) was used for imaging. Nuclei were counterstained with hematoxylin. Incubation in the absence of primary antibodies served as a negative control.

### 2.6. Semi-Quantitative and Quantitative Scoring of the Immunohistochemistry Study

The expression of markers was assessed by both quantitative and semi-quantitative methods.

**Semi-Quantitative Scoring of the Immunohistochemistry Study.** The immunoreactivity score was determined by the intensity and distribution of the specific stain and expressed as ‘+++’–strong diffuse positive, ‘++’–moderate diffuse positive, ‘+’–mild or moderate focal positive, ‘+−‘– weak positive, or ‘−’–no stain. All immunohistochemical slides were independently evaluated by three investigators.

**Evaluation of Odontogenic and Osteogenic Expression.** The number of antigen-positive cells was determined in three images of random fields of view obtained with a magnification of the microscope lens of 20× by means of the ImageJ1.54m software (Wayne Rasband, National Institute of Health, Bethesda, MA, USA). The approximate number of total cells contained in the randomly selected field for 3D cultures ranged from 100 to 200. The percentage of cells that expressed the osteogenic or odontogenic differentiation marker was calculated by dividing the number of positively stained cells by the total number of cells per field of view multiplied by 100.

### 2.7. ELISA Assay

The supernatants were collected on day 14 before fixation of 3D cultured cells and frozen at -70 °C. ELISA Kits (Cloud-Clone Corp.) for TGF-β1, BMP-2, Osteopontin (OPN), and Osteocalcin (OC) were used. The ELISA procedures were performed according to the manufacturer’s instructions. All samples (three from each group) were analyzed in duplicate; as a result, n=6 for each group. The absorbance was measured at 450 nm by a multifunctional plate reader (Multiskan FC Microplate Photometer, Thermo Fisher Scientific, Katy, TX, USA).

### 2.8. Statistical Analysis

Statistical data processing was performed using a one-way analysis of variance (ANOVA) with Dunnett’s multiple comparison test with GraphPad Prism v.10.4.0(621) software (GraphPad Software, Boston, MA, USA). The results of the quantitative morphometric analysis are represented as the mean ± standard error of the mean (SEM), n = 3. The results of the ELISA analysis are represented as box-plot graphs in the Tukey style, n = 6. A change was considered significant at *p* < 0.05.

## 3. Results

**Figure 1:**
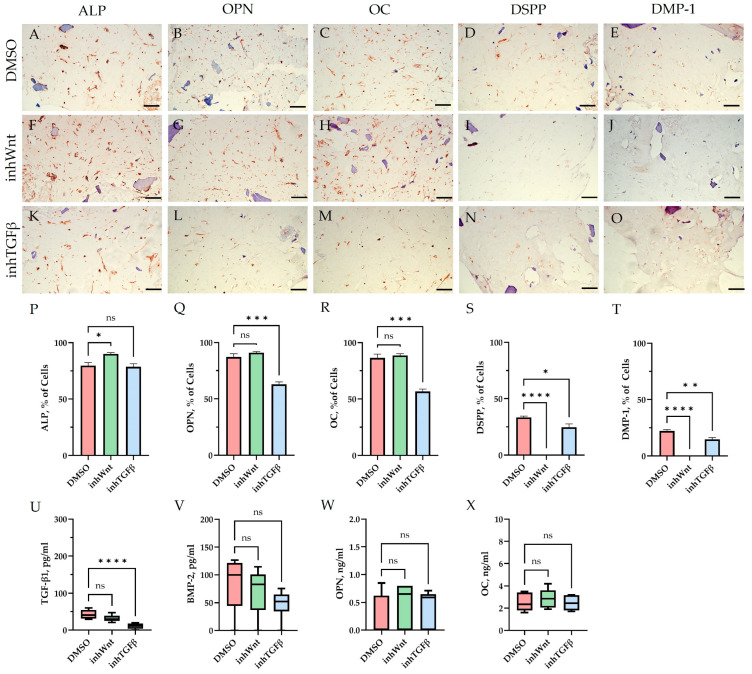
Evaluation of the expression of different markers (**A**–**T**) and ELISA (**U**–**X**) in the control group (DMSO) and in groups after inhibition of the Wnt signaling pathway (inhWnt) and TGF-β signaling pathway (inhTGFβ) without the addition of ECM components: (**A**–**O**) immunoperoxidase staining. Expression of osteogenic markers (ALP (**A**,**F**,**K**), OPN (**B**,**G**,**L**), and OC (**C**,**H**,**M**)) and odontogenic markers (DSPP (**D**,**I**,**N**) and DMP-1 (**E**,**J**,**O**)). The nuclei were counterstained with hematoxylin. Scale bars, 100 µm; (**P**–**T**) morphometric analysis of the percentage of cells stained with ALP, OPN, OC, DSPP, and DMP-1 in different groups; n = 3. Values were expressed as mean ± SEM. ns, non-significant; * *p*  <  0.05; ** *p*  <  0.01; *** *p*  <  0.001; **** *p*  <  0.0001; (**U**–**X**) secretion of TGF-β1 (**U**), BMP-2 (**V**), OPN (**W**), and OC (**X**) by PDLSCs. Boxplots, n = 6. ns, non-significant; **** *p* < 0.0001.

Immunohistochemical results in the control group (DMSO), when PDLSCs were cultured in decellularized scaffolds under 3D conditions for 14 days without inhibition of the Wnt and TGF-β signaling pathways and without the addition of ECM components, as in the previous study [16], showed spontaneous differentiation of PDLSCs in the osteogenic direction (Figure 1A–C; Table 1) and odontogenic direction (Figure 1D,E; Table 1). ELISA of the supernatants revealed the secretion of TGF-β1–42.63 ± 11.63 pg/mL, BMP-2–82.7 ± 45.29 pg/mL, OPN–0.26 ± 0.36 ng/mL, and OC–2.5 ± 0.80 ng/mL (Figure 1U–X).

**Figure 2:**
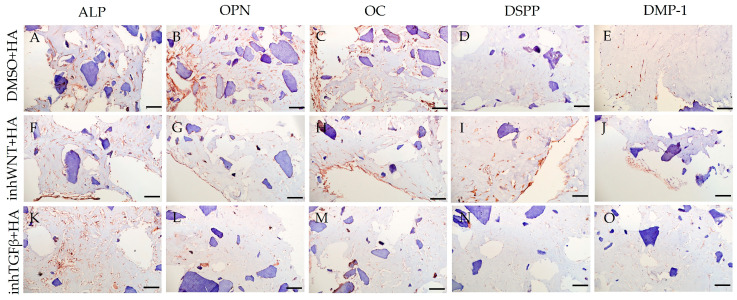
Immunoperoxidase staining. Expression of osteogenic markers (ALP (**A**,**F**,**K**), OPN (**B**,**G**,**L**), and OC (**C**,**H**,**M**)) and odontogenic markers (DSPP (**D**,**I**,**N**) and DMP-1 (**E**,**J**,**O**)) in PDLSCs with the addition of hyaluronic acid (HA). The nuclei were counterstained with hematoxylin. Scale bars, 100 µm: (**A**–**E**) plus adjuvant of DMSO; (**F**–**J**) under inhibition of the Wnt signaling pathway (inhWnt); (**K**–**O**) under inhibition of the TGF-β signaling pathway (inhTGFβ).

**Figure 3:**
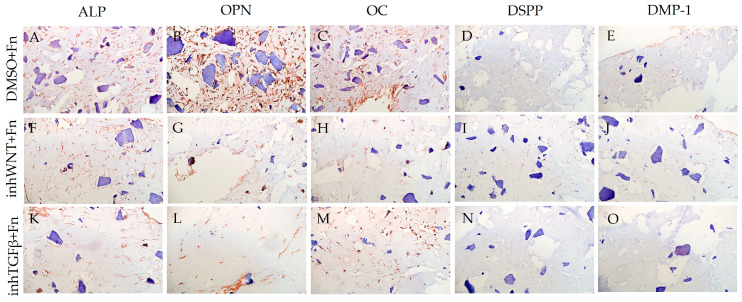
Immunoperoxidase staining. Expression of osteogenic markers (ALP (**A**,**F**,**K**), OPN (**B**,**G**,**L**), and OC (**C**,**H**,**M**)) and odontogenic markers (DSPP (**D**,**I**,**N**) and DMP-1 (**E**,**J**,**O**)) in PDLSCs with the addition of fibronectin (Fn). The nuclei were counterstained with hematoxylin. Scale bars, 100 µm: (**A**–**E**) plus adjuvant of DMSO; (**F**–**J**) under inhibition of the Wnt signaling pathway (inhWnt); (**K**–**O**) under inhibition of the TGF-β signaling pathway (inhTGFβ).

**Figure 4:**
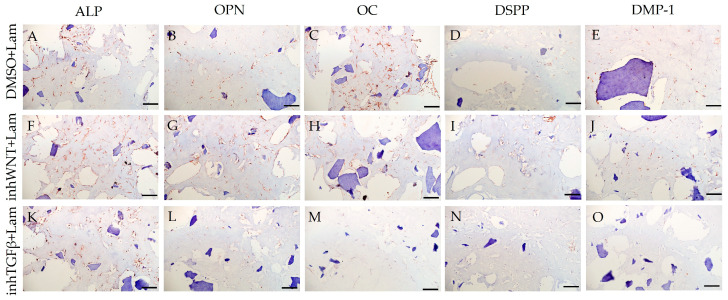
Immunoperoxidase staining. Expression of osteogenic markers (ALP (**A,F,K**), OPN (**B,G,L**), and OC (**C,H,M**)) and odontogenic markers (DSPP (**D,I,N**) and DMP-1 (**E,J,O**)) in PDLSCs with the addition of laminin (Lam). The nuclei were counterstained with hematoxylin. Scale bars, 100 µm: (**A**–**E**) plus adjuvant of DMSO; (**F**–**J**) under inhibition of the Wnt signaling pathway (inhWnt); (**K**–**O**) under inhibition of the TGF-β signaling pathway (inhTGFβ).

**Figure 5:**
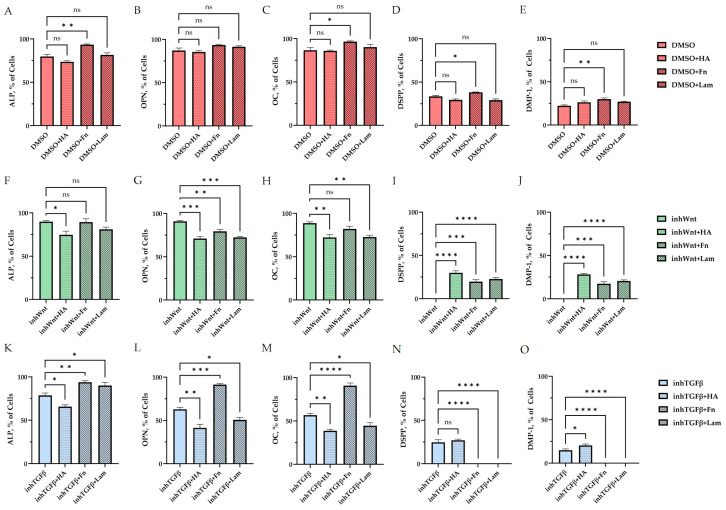
Morphometric analysis of the percentage of cells stained against ALP (**A**,**F**,**K**), OPN (**B**,**G**,**L**), OC (**C**,**H**,**M**), DSPP (**D**,**I**,**N**), and DMP-1 (**E**,**J**,**O**) (**A**–**E**) without inhibition of the Wnt and TGF-β signaling pathways and with the addition of ECM components (hyaluronic acid (HA), fibronectin (Fn), and laminin (Lam)) in comparison to the control group (DMSO); (**F**–**J**) after inhibition of the Wnt signaling pathway (inhWnt) and the addition of components of the ECM (HA, Fn, Lam) in comparison to inhWnt; (**K**–**O**) after inhibition of the TGF-β signaling pathway (inhTGFβ) and the addition of components of the ECM (HA, Fn, Lam) in comparison to inhTGFβ; n = 3. Values are expressed as mean ± SEM. ns, non-significant; * *p*  <  0.05; ** *p*  <  0.01; *** *p*  <  0.001; **** *p*  <  0.0001.

**Figure 6:**
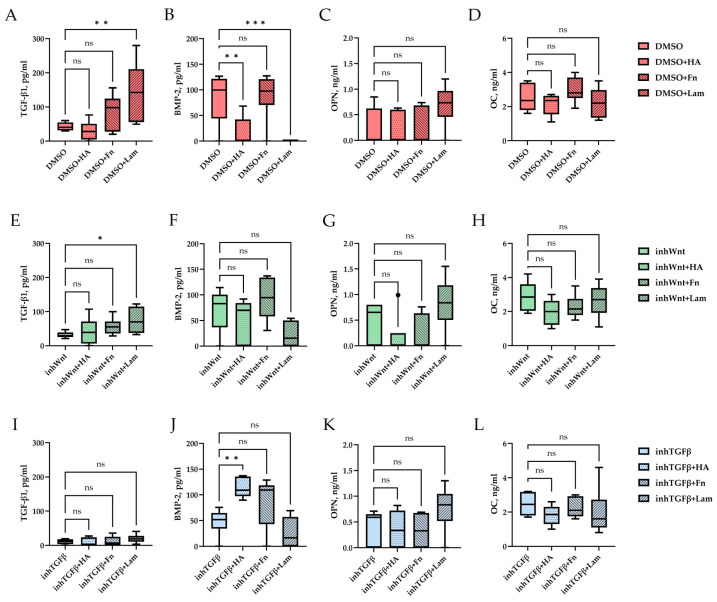
ELISA. Secretion of TGF-β1 (**A,E,I**), BMP-2 (**B,F,J**), OPN (**C,G,K**), and OC (**D,H,L**) by PDLSCs under different conditions: (**A**–**D**) in a model without inhibition of the Wnt and TGF-β signaling pathways and with the addition of ECM components (hyaluronic acid (HA), fibronectin (Fn), and laminin (Lam)) compared to the control group (DMSO); (**E**–**H**) in a model with inhibition of the Wnt signaling pathway (inhWnt) and the addition of components of the ECM (HA, Fn, Lam) compared to inhWnt; (**I**–**L**) in a model with inhibition of the TGF-β signaling pathway (inhTGFβ) and the addition of components of the ECM (HA, Fn, Lam) compared to inhTGFβ; n = 6. Box-plot graphs in the Tukey style; ns, non-significant; * *p*  <  0.05; ** *p*  <  0.01; *** *p*  <  0.001.

Expression of osteogenic and odontogenic markers was also detected in the presence of one or another ECM components (HA (Figure 2A–E; Table 1), Fn (Figure 3A–E; Table 1), and Lam (Figure 4A–E; Table 1)). The addition of Fn primarily promoted the expression of osteogenic and odontogenic markers (Figure 5A–E). However, the ELISA results did not reveal statistically significant differences in the levels of the osteogenic markers OPN and OC when the ECM components were added. The addition of Lam without inhibitors significantly increased TGF-β1 production in PDLSCs (Figure 6A), while HA and Fn did not change it (Figure 6A). At the same time, HA and Lam reduced BMP-2 production in the PDLSCs (Figure 6B), and Fn did not have a noticeable effect on its secretion (Figure 6B).

Blocking the Wnt signaling pathway in the group without the addition of components of the ECM revealed suppressed expression of odontogenic markers in the PDLSCs (Figure 1I,J,S,T, Table 1), with little or no effect on the expression of osteogenic markers (Figure 1F–H,P–R, Table 1) compared to the control group. The secretion of TGF-β1 and BMP-2 did not change significantly (Figure 1U,V).

The addition of HA, Fn and Lam while blocking the canonical Wnt signaling pathway contributed to the restoration of the expression of odontogenic markers in the PDLSCs (Figure 2I,J; Figure 3I,J; Figure 4I,J, respectively; Figure 5I,J; Table 1), slightly reducing the expression of osteogenic differentiation markers (Figure 2F–H; Figure 3F–H; Figure 4F–H, respectively; Figure 5F–H; Table 1) compared to the inhWnt group. However, the results of the ELISA did not reveal statistically significant differences in the levels of the osteogenic markers OPN (Figure 6G) and OC (Figure 6H) when ECM components were added. Lam increased TGF-β1 secretion by the PDLSCs (Figure 6E), while HA and Fn did not change it (Figure 6E). The secretion of BMP-2 by PDLSCs was not altered (Figure 6F).

In contrast to inhibition of the Wnt signaling pathway, inhibition of the TGF-β signaling pathway significantly suppressed the expression of osteogenic markers in the PDLSCs (Figure 1L,M,Q,R; Table 1) and slightly reduced the expression of odontogenic differentiation markers (Figure 1N,O,S,T; Table 1). TGF-β1 was reduced (Figure 1U), but BMP-2 secretion was not significantly altered (Figure 1V). The ELISA results did not reveal statistically significant differences in OPN and OC secretion when the Wnt and TGF-β signaling pathways were blocked (Figure 1W,X). Fn promoted the increase in osteogenic markers in the PDLSCs (Figure 3K–M and Figure 5K–M; Table 1), while HA and Lam decreased them compared to the inhTGFβ group (Figure 2K–M; Figure 4K–M, respectively; Figure 5K–M; Table 1). At the same time, Fn and Lam suppressed the expression of odontogenic differentiation markers (Figure 3N,O; Figure 4N,O, respectively; 5N,O; Table 1), while the addition of HA preserved it (Figure 2N,O and Figure 5N,O; Table 1). The ELISA results did not reveal statistically significant differences in the secretion of the osteogenic markers OPN (Figure 6K) and OC (Figure 6L) when ECM components were added. The amount of TGF-β1 did not change significantly in all groups. At the same time, incubation with HA increased BMP-2 production under inhibition of the TGF-β signaling pathway (Figure 6J).

Thus, under conditions of violation of the Wnt/β-catenin and TGF-β signaling pathways, a multidirectional effect of the ECM components on the expression of osteogenic and odontogenic markers in PDLSCs was established.

## 4. Discussion

A distinctive feature of inflammatory/infectious processes in periodontitis is the increased production of cytokines, proteases, and other pro-inflammatory mediators that disrupt the homeostasis of periodontal tissues [5,18,19]. Disturbances in signaling pathways caused by changes in the microenvironment during inflammatory processes can lead to an imbalance of immune reactions and disorders in the differentiation of resident stem cells migrating to the lesion. The degradation of ECM also contributes to impaired signaling in stromal cell interactions. Therefore, in addition to antibiotic therapy, the main approach in the treatment of periodontitis is the use of biomatrices, which ensure the restoration of key matrix signals that ensure the recruitment of endogenous stem cells and their differentiation.

The application of dECM is an attractive strategy in the repair of damaged tissues [20]. The architectonics of the ECM components that contain matrix-associated factors form a microenvironment that promotes the homing of resident stem cells to the damage zone [21].

The combination of dTM and dPDL in collagen I hydrogel induces spontaneous differentiation of PDLSCs in osteogenic and odontogenic directions, and the addition of Fn further enhances the expression of both osteogenic and odontogenic markers. A network of Fn and type I collagen microfibrils is known to facilitate the formation of latent forms of TGF-β and BMP by serving as their reservoir in the bone and cartilage matrix [22].

In addition, the Wnt/β-catenin and TGF-β signaling pathways are known to be essential for periodontal tissue development. Both the canonical WNT/β-catenin and TGFβ pathway stimulate each other via the Smad and non-Smad pathways, such as signaling of phosphatidylinositol-3-kinase/serine/threonine kinase (PI3K/Akt), which regulates periodontal tissue differentiation [23]. Savithri et al. have shown that there is a gradient of Wnt/β-catenin activity throughout PDL with higher activity at the cement/PDL interface, which correlates with increased cell proliferation. On the other hand, lower Wnt/β-catenin activity was found at the interface between PDL and alveolar bone, which corresponds to increased differentiation towards an osteogenic clone. This suggests that the effects of the Wnt/β-catenin signaling pathway depend on the spatial arrangement of the cells, which affects both their proliferation and differentiation in PDL [24].

In our study, we attempted to investigate the effect of Wnt/β-catenin and TGF-β signaling pathways on the differentiation of PDLSCs under homeostasis-disruptive conditions arising from periodontal diseases. The small molecule IWP-2 was used to inhibit the Wnt/β-catenin signaling pathway and block cell proliferation and differentiation through the canonical pathway. To block the TGF-β signaling pathway, a small molecule A 83-01 was used, which inhibits the type I receptor TGF-β, but does not affect the receptors of bone morphogenetic proteins. The collagen hydrogel containing dECM was supplemented with individual ECM components as potential constituents of functional bioscaffolds.

The results of the study demonstrated that the addition of ECM components restored the expression of odontogenic differentiation markers in PDLSCs, which was absent when the canonical Wnt signaling pathway was inhibited. At the same time, ECM components had a multidirectional effect on the secretion of TGF-β1 and BMP-2. Based on the results obtained, it may be suggested that activation of the Wnt/β-catenin signaling pathway is necessary for the odontogenic differentiation of PDLSCs. The results of other researchers demonstrate contradictory data on this issue [8,9,12]. The determination of TGF-β1 and BMP-2 levels by ELISA in our study did not reveal a significant role for these factors in the expression of markers of odontogenic differentiation in PDLSCs, which may be due to the presence of these factors in decellularized matrices of the tooth and periodontal ligament [15,25].

The addition of Fn and Lam while inhibiting the TGF-β signaling pathway suppressed the expression of odontogenic markers in the PDLSCs, exerting a multidirectional effect on BMP-2 secretion. Inhibition of the TGF-β1 signaling pathway is known to significantly inhibit osteogenic differentiation of PDLSCs through competition with BMP-2 [26,27]. At the same time, several researchers note that cementoblastic differentiation of PDLSCs is induced by blocking TGF-β1 signaling [3]. For example, overexpression of Runx2, the master regulator of osteoblast differentiation into odontoblasts, has been shown to inhibit terminal odontogenic differentiation and convert them to an osteogenic phenotype [28]. Since the Wnt signaling pathway is an evolutionarily conserved intracellular signaling that plays an important role in osteogenic cell differentiation, it is likely that TGF-β may inhibit Wnt signaling by reducing Runx2 expression. This may explain how the TGF-β signaling pathway regulates the osteo/odontogenic phenotype of mature root odontoblasts. However, the molecular mechanisms underlying these regulations need to be further clarified [28]. It can be assumed that Fn and Lam inhibit the expression of odontogenic differentiation markers in PDLSCs, activating Wnt/ β-catenin signaling through the integrin a9b1-FAK-MAPK/ERK pathway [29,30]. On the basis of these data, we believe that it is not advisable to include Fn and Lam in the composition of multicomponent scaffolds in the treatment of periodontitis.

The presence of HA without inhibition of thr Wnt/β-catenin and TGF-β signaling pathways depressed odontoblastic differentiation [31] and blocked BMP-2 production, which is consistent with previously published data [32], while the addition of HA upon the inhibition of thr TGF-β signaling pathway improved BMP-2 secretion, maintaining/restoring the expression of markers of odontogenic differentiation in PDLSCs. According to the literature, HA stimulates the TGF-β/BMP signaling pathway and promotes the secretion of TGF-β1 and TMR, and suppression of this signaling pathway inhibits the secretion of these proteins [33]. Since in our study we used a molecule that inhibits type I receptor TGF-β but does not affect BMP receptors, BMP-2 secretion was enhanced in the presence of HA. Consequently, HA can be considered a component that enhances/restores the osteogenic and odontogenic differentiation of PDLSCs.

## 5. Conclusions

Thus, the results demonstrated that when the Wnt/β-catenin and TGF-β signaling pathways that develop during inflammatory processes in the periodontium are disrupted, disorders in the differentiation of PDLSCs occur, preventing periodontal tissue regeneration. This should be taken into account when developing multicomponent scaffolds that recapitulate the microenvironment at the endogenic regeneration of the periodontium. Since the HA addition contributed to the maintenance of both the osteogenic and odontogenic differentiation of PDLSCs, the inclusion of HA as one of the key components of scaffolds may enhance the therapeutic effect of such biomaterials.

## Figures and Tables

**Table 1:** Semi-quantitative estimation of odontogenic and osteogenic markers in PDLSCs under different culture conditions.

Marker	DMSO	DMSO +HA	DMSO +Fn	DMSO +Lam	inhWnt	inhWnt +HA	inhWnt +Fn	inhWnt +Lam	inhTGFβ	inhTGFβ +HA	inhTGFβ +Fn	inhTGFβ +Lam
ALP	+++	++	+++	++	++	+ /++	+	+/++	+	+	+	+
OPN	++	++	+++	++	++	+	++	+/++	+	+	+	+
OC	++	++	+++	++	++	+	+/++	+/++	+	+	++	+
DSPP	+	+	+	+	−	+	+	+	+/+−	+	−	−
DMP-1	+	+	+	+	−	+	+	+	+/+−	+	−	−

‘+++’–strong diffuse positive, ‘++’–moderate diffuse positive, ‘+’–mild or moderate focal positive, ‘+−‘—weak positive, ‘−’—no stain.

## Data Availability

The data presented in this study are available on request from the corresponding author. The data are not publicly available due to legal issues.

## Abbreviations

The following abbreviations are used in this manuscript:

TGF-β	Transforming growth factor beta
BMP	Bone morphogenetic protein
PDL	Periodontal ligament
PDLSCs	Periodontal ligament stem cells
dPDL	Decellularized periodontal ligament
dTM	Decellularized tooth matrix
ECM	Extracellular matrix
dECM	Decellularized ECM
Fn	Fibronectin
Lam	Laminin
HA	Hyaluronic acid
Col I	Collagen I hydrogel
inhWnt	Wnt/β-catenin inhibitor
inhTGFβ	TGF-β inhibitor
DMSO	Dimethylsulfoxide
ALP	Alkaline phosphatase
OPN	Osteopontin
OC	Osteocalcin
DSPP	Dentin sialophosphoprotein
DMP-1	Dentin matrix acidic phosphoprotein 1
